# Task-shifting impact of introducing a pilot community health worker cadre into Zambia’s public sector health workforce

**DOI:** 10.1371/journal.pone.0181740

**Published:** 2017-08-02

**Authors:** Brett Keller, Elizabeth McCarthy, Kathryn Bradford Vosburg, Mutinta Musonda, Jere Mwila, Jan Willem van den Broek, Fiona J. Walsh

**Affiliations:** 1 Applied Analytics, Clinton Health Access Initiative, Dar es Salaam, Tanzania; 2 Applied Analytics, Clinton Health Access Initiative, Lusaka, Zambia; 3 Human Resources for Health, Clinton Health Access Initiative, Lusaka, Zambia; 4 Cabinet Office, Government of Zambia, Lusaka, Zambia; 5 Clinton Health Access Initiative, Lusaka, Zambia; 6 Applied Analytics, Clinton Health Access Initiative, Boston, United States; London School of Hygiene & Tropical Medicine, UNITED KINGDOM

## Abstract

**Background:**

The Zambia Ministry of Health (MOH) recruited and trained a new cadre of *Community Health Assistants* (CHAs) as part of its National Community Health Strategy. The inaugural class of 307 CHAs completed one year of training in July 2012 and deployed to their communities.

**Methods:**

The impact of the CHA program on the volume and type of health services provided at health posts and their respective referral health centers was measured with a non-randomized difference-in-differences design. Monthly health service provision data was collected for 12 months before and after CHA deployment at 8 health posts along with 8 referral health centers. The analysis controlled for seasonality, changes in non-CHA staffing, and periodic regional child health campaigns, and used facility-level fixed effects.

**Results:**

Deploying two CHAs to a health post did not lead to a statistically-discernible increase in services at the intervention facilities. Health services provided at referral health centers increased by 697.9 services per month (95% CI: 131.4 to 1,264.3, p = .016), and combined services (at health posts and referral health centers) increased by 848.6 services per month (95% CI: 178.2 to 1,519.1, p = .013).

**Conclusion:**

In this pilot, the addition of CHAs in rural areas increased health service provision at referral health facilities and at facilities overall, shifting the burden of basic health services away from more highly trained health workers. Shifting tasks to lesser-trained, less-expensive cadres like the CHAs, policymakers can rapidly improve access to care with constrained budgets. Evaluations measuring the direct impact of lower level cadres without accounting for task-shifting may underestimate their contribution to the health workforce.

## Introduction

Many low- to middle-income countries are currently struggling to close the widening gap between the population’s health demands and the number of health workers. To address these human resources for health (HRH) shortages, several initiatives have suggested task shifting to community health workers (CHWs), with task shifting defined as “optimizing the roles of less specialized health workers” [1]. CHWs are typically selected from within communities to provide basic health services such as community education, diagnosing common diseases, and providing limited medical treatment [2].

Evidence suggests that CHWs, who typically cost less to train and employ than other cadres, are capable of providing high-quality basic health care services. For example, a review of the effectiveness of CHWs for the delivery of HIV services found that delegating specific tasks, such as HIV and nutrition education, to cadres of CHWs with limited training can increase access to HIV services, particularly in rural areas and underserved communities [2]. A 2013 review by Gilmore and McAuliffe [3] found evidence that CHWs are also effective in delivering preventive and educational interventions and increasing health-seeking behaviors. Similarly, Perry et al. [4] found that CHWs can be effective in improving population health as an integral part of comprehensive health systems.

Yet, limited evidence exists around *how* CHWs shift service delivery within health systems. One limitation of the existing literature on task shifting is that many studies compare newly introduced cadres to traditional cadres, rather than to whether care would have been provided at all in the absence of the new cadre [5]. Given that newly introduced health workers may increase health service delivery either directly, by providing the services, or indirectly, by freeing up health worker time to provide more specialized or complicated services, evaluations of the impact of CHWs should measure these broader effects.

### Building a community health worker strategy in Zambia

Zambia is a lower-middle income and predominantly rural country in sub-Saharan Africa with poor but improving health outcomes [6]. From 2007 to 2014 the maternal mortality ratio declined from 591 to 398 deaths per 100,000 live births and the under 5 mortality rate declined from 119 to 75 deaths per 1,000 live births [7] [8]. While mortality has declined, a large shortfall in health workers has been cited by the Government of Zambia as one of its greatest challenges to further expanding equitable access to essential health services [9]. In May 2012, Zambia only had 12.6 doctors, nurses and midwives per 10,000 population, far short of the WHO-recommended minimum of 5.5 doctors and 17.3 nurses per 10,000 population [10]. The national health workforce shortage crisis is particularly acute in rural areas, where 60.5% of Zambia’s population resides [11].

In 2010, the Ministry of Health (MOH) launched a National Community Health Worker Strategy with the goal of deploying an effective, well-trained, and motivated cadre to improve maternal and child health in rural areas. The scope of work for these Community Health Assistants (CHAs) includes preventative and basic curative services with complicated patient cases referred from the community-level to higher-level health workers based at nearby health facilities [12]. CHAs are nominated by their communities and return home to start providing care after one year of formal training. CHAs are meant to split their time between conducting community outreach and providing services in Health Posts, the most basic level of facility in Zambia. The pilot class of 307 CHAs graduated in July 2012 and deployed back to their communities. The training program was accredited by the University of Zambia, and–per their regulations–all students have to take a comprehensive final exam at the end of the studies in addition to their regular assessments after each training module. Once students pass their final exam, they can apply for licensing by the Health Professionals Council of Zambia, which regulated the CHA’s scope of work and practice. All students in the first cohort included in this study passed their final exam and were licensed before officially deployed by MOH back to their home communities.

While the initial funding for CHA remuneration was supported by development aid partners, the CHAs were transitioned to the public sector payroll and thus fully integrated into the public sector health workforce in December 2013. The CHA scope of work and challenges in their initial community deployments are described in detail in by Shelley et al. in their findings of a 2012–2013 process evaluation of the CHA program to guide future scale-up decisions [12].

**Fig 1:**
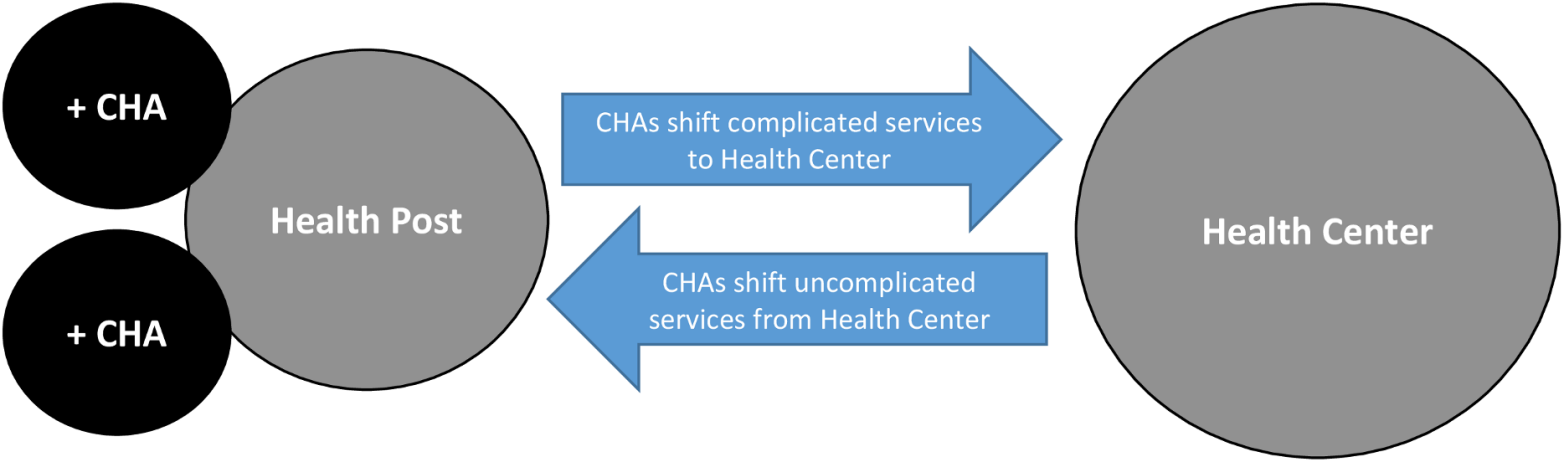
Schematic of CHA task-shifting effects at rural health posts and health centers in Zambia.

With this task-shifting evaluation, we aimed to measure the impact of the pilot CHA class on the health system during the first year of CHA deployment. As illustrated in Fig 1, the evaluation tested the hypothesis that CHA deployment to Health Posts would increase the provision of uncomplicated services at the Health Posts and lead to an increase in complicated services provided at referral Health Centers as health worker time was freed up.

Services provided at health facilities were categorized as either uncomplicated—those within the CHA scope of work, including preventive services and outpatient department under 5 visits—and complicated services which CHAs are not trained to provide, such as initiating antiretroviral treatment. The personnel cost of CHAs including training and salaries were documented and combined with CHA impacts to estimate cost-effectiveness.

## Methods

This research was approved by the University of Zambia Biomedical Research Ethics Committee (UNZAREC). This difference-in-difference (DiD) evaluation compared service delivery volumes at comparison and intervention facilities before and after CHA deployment, assessing the direct impact of CHA deployment to Health Posts as well as the indirect effect of the CHAs’ arrival at Health Posts on their referral Health Centers. CHA assignment was not randomized because the study was designed after CHA selection but prior to deployment, and CHAs are deployed to the communities from which they were selected. Data from 8 Health Posts and 8 Health Centers were analyzed.

### Site selection

For the pilot phase, MOH limited CHA program participation to rural and hard to reach communities, resulting in recruitment from seven Provinces and 48 Districts. The CHAs were selected by their own communities, and are currently being trained for one year before being deployed back to their rural communities.

The MOH and the Clinton Health Access Initiative (CHAI) engaged provincial and district health staff to select Health Posts to participate in the pilot that were 1) hard to reach, 2) close to referral Health Centers, 3) lower-performing in terms of number of health services in the health management information system (HMIS), and 4) minimally staffed. This selection process was pragmatic given the need to balance contradicting criteria (remoteness and low performance vs. closeness to referral centers). One hundred and sixty-one Health Posts were selected for pilot deployment of CHAs. For this evaluation four rural districts with at least two Health Posts were selected via stratified purposive sampling. One CHA pilot Health Posts and one comparison Health Post were selected in each district. Sample sites are described in Table 1. As seen at Kaseba, Mulaushi, and Nabukowa Health Post, the average monthly staffing at a facility in the baseline or endline period was less than 1 if a health worker was not deployed at the facility for the entire year.

**Table 1:** Comparison and intervention sites by province, district, and staffing.

Province	District	Health Post	Assignment	Average monthly staffing during baseline period	Average monthly staffing during endline period
Central	Serenje	Kaseba	Comparison	0.7 nurse, 0.3 midwife	1 nurse
Central	Serenje	Mulaushi	Intervention	0.5 nurse	1 nurse, 2 CHAs
Southern	Sinazongwe	Nabukowa	Comparison	none	0.7 nurse, 0.7 midwife
Southern	Sinazongwe	Muuka	Intervention	none	2 CHAs
Eastern	Chadiza	Chanjowe	Comparison	1 midwife	1 clinical officer, 1 nurse, 1 midwife
Eastern	Chadiza	Sinalo	Intervention	1 nurse	1 nurse, 2 CHAs
Northern	Luwingu	Lufubu	Comparison	none	none
Northern	Luwingu	Laurenti-Chita	Intervention	none	2 CHAs

Two of the four intervention Health Posts, as well as two of the four comparison Health Posts, had other clinically-trained staff (nurses or midwives) at baseline. The eight Health Centers to which the eight study Health Posts refer patients were also selected for the study to measure the impact of CHA deployment at Health Posts on task-shifting at referral facilities. One comparison site, Kaseba Health Post, was substituted into the study to replace a comparison site to which CHAs were deployed.

### Data sources

Data were abstracted from Health Post and Health Center facility registers. 12 months each of baseline data (May 2011 to April 2012) and post-CHA deployment endline data (September 2012 to August 2013) were collected, with a gap between the periods due to administrative delays in initial CHA deployment. Health services were categorized as uncomplicated if included in CHA scope of work. Services not included in the CHA scope of work were categorized as complicated.

Reasons for missing data and changes in facility staffing were discussed with facility and district staff. Cost data including CHA salaries and training costs were obtained from discussions with MOH. Training costs were amortized over 30 years to generate a monthly cost for CHA deployment combining salary and training costs.

### Data analysis

Data were analyzed using Stata (Version 12.1 2013; College Station, TX). The effect of CHAs was estimated for outcome measures: total service provision, complicated service provision, uncomplicated service provision, and patient visits. Further analyses were conducted on four specific services, including under 5 outpatient department (OPD) visits, over 5 OPD visits, first antenatal care (ANC1) visits, and facility deliveries.

The main analysis used a DiD specification to control for time-invariant facility-level characteristics. The analysis controlled for the impact of staffing changes among other types of health workers, seasonality, and special service campaigns. Fixed effects were employed to maximize statistical power given constraints on the number of facilities with data collection. The main DiD equation was:

$$Y=\beta_{0}+\beta_{1}\alpha +\beta_{2}\gamma +\beta_{3}\delta +\beta_{4}\zeta +\beta_{5}\eta +\beta_{6}\vartheta +\beta_{7}\lambda +\varepsilon ,$$

where *Y* = outcome measure for facility and month, α = baseline versus endline dummy, γ = intervention dummy, *δ* = *α* x *γ*, the DiD indicator, *ζ =* dummy for each health facility (facility fixed effects), *η* = control for the total number of non-CHA health staff (clinical officers, nurses, midwives) at the facility that month, *ϑ* = Child Health Day campaign dummy, and *λ =* wet (November to April) versus dry season dummy. The campaign dummy was included in the main DiD specification to control for the impact of the influx of health workers during period Child Health Day campaigns (verified through administrative data) on non-campaign services, and high-volume campaign services (Vitamin A and deworming) were excluded from the total services outcome.

In total, 384 months of data were collected (12 months at baseline and endline for 16 facilities). Missing data was a challenge: Nabukowa Health Post was included in the study based on district data but was found to be missing all 24 months of service delivery data due to a termite infestation at the facility and is effectively excluded from the analysis This excluded facility did not differ from included facilities in terms of available staffing, but given the missing data it cannot be tested for differences in service provision volumes. Across the other fifteen facilities, 12 complete facility-months of data were missing (3 months of data at Muuka HP; 2 months at Buleya Malima HC, Kaseba HP, Muchinka HC, and Mulaushi HP; and 1 month of data at Siatwinda HC). Overall, facility-months with missing data did not differ from facility-months with available data in terms of seasonality, staffing, or intervention status. For all time periods, facility-level missing data on patient visits and service delivery were imputed via mean substitution from the same facility, averaging across months when the same facility had identical intervention status (before vs. after), staffing, and seasonality.

## Results

The results of this evaluation include the direct impact of CHA deployment on health service provision and patient volumes at the Health Posts to which they were deployed, the indirect impact of CHA deployment to Health Posts on health service provision and patient volumes at referral Health Centers, the combined direct and indirect impact (the overall task shifting effect), and the cost-effectiveness of the combined impact. CHAs were deployed in pairs, and all results in this section are presented in terms of the effect of deploying *two* CHAs to a Health Post unless otherwise noted. Only eight Health Posts were included in this evaluation, resulting in large standard errors and confidence intervals around DiD effects. Results with p < .05 are reported as statistically discernible.

### Direct impact on service volumes at Health Posts

The direct impact of CHA deployment to Health Posts is shown in Table 2.

**Table 2:** Direct impact of CHA deployment at health posts.

Outcome	Average monthly services at comparison sites		Average monthly services at intervention sites			Difference-in-Difference with controls for staffing, seasonality, and campaigns	
	Baseline	Endline	Baseline	Endline	P	Effect (95% CI)	p
All non-campaign services	2,199	2,958	1,175	2,212	0.128	+164.5 (-167.2, 496.2)	0.329
Complicated services	561	733	456	551	0.572	+75.2 (-54.2, 204.6)	0.253
Uncomplicated services	1,639	2,225	833	1,661	0.096	+99.5 (-144.5, 343.5)	0.422
Specific services:							
All outpatient visits	1,062	1,410	753	1,420	0.691	-89.7 (-291.9, 112.6)	0.382
Outpatient visits under 5	505	557	345	680	0.087	+38.9 (-72.3, 145.0)	0.490
Outpatient visits over 5	557	853	407	741	0.264	-128.5 (-231.7, -25.3)	0.015*
Antenatal care - 1st visit	23.8	20.8	13.5	17.7	0.012	+9.2 (1.1, 17.2)	0.026*
Antenatal care - 1st visit before 20 weeks						+2.2 (-0.7, 5.1)	0.209
Antenatal care - 1st visit after 20 week						+6.6 (2.7, 10.4)	0.005
Facility delivery	6.1	11.3	7.2	8.1	0.520	-0.9 (-4.0, 2.3)	0.578
Patient visits	464	641	193	339	0.373	+74.6 (-58.3, 207.5)	0.269

* = significant at the 0.05 level

Health Posts that received two CHAs delivered a non-statistically discernible 164.5 additional services per month (95% confidence interval [CI]: -167.2 to 496.2, p = 0.329) compared to Health Posts that did not receive CHAs. The changes in complicated and uncomplicated health services and in patient visits were not statistically discernible. The only statistically discernible changes in specific service provision were for over-five OPD visits, which decreased by 128.5 visits (31.6% decrease) per month (95% CI: -231.7 to 25.3, p = 0.015), and for ANC1 visits which increased by 9.2 per month (68.1% increase, 95% CI: 1.1 to 17.2, p = 0.026).

### Indirect impact on service volumes at Health Centers

The indirect impact of CHA deployment to Health Posts was measured at the Health Centers to which comparison and intervention Health Posts refer patients. The DiD results for this impact are summarized in Table 3.

**Table 3:** Indirect impact of CHA deployment at referral health centers.

Outcome	Average monthly services at comparison sites		Average monthly services at intervention sites			Difference-in-Difference with controls for staffing, seasonality, and campaigns	
	Baseline	Endline	Baseline	Endline	p	Effect (95% CI)	p
All non-campaign services	4,939	6,547	3,064	5,004	0.008*	+697.9 (131.4, 1264.3)	0.016*
Complicated services	1,684	2,484	1,100	1,776	0.613	+358.1 (166.1, 550.2)	<0.001*
Uncomplicated services	3,255	4,063	1,964	3,228	0.003	+339.7 (-120.4, 799.9)	0.147
Specific services:							
All outpatient visits	1,000	1,478	1,046	1,729	0.284	-122.0 (-393.1, 149.1)	0.376
Outpatient visits under 5	530	802	417	596	0.391	-192.2 (-292.9, -91.6)	<0.001*
Outpatient visits over 5	469	676	630	1,134	0.064	+70.2 (-150.3, 290.8)	0.531
Antenatal care - 1st visit	39.7	45.5	38.6	41.2	0.686	+3.2 (-6.7, 13.2)	0.523
Facility delivery	24.2	23.7	15.3	22.6	0.656	-0.7 (-8.6, 7.3)	0.872
Patient visits	1,117	1,193	649	1,287	<0.001	+580.1 (312.6, 847.5)	<0.001*

* = significant at the 0.05 level

The addition of two CHAs to a Health Post led to an increase in all health service provision of 697.9 services per month (22.8% increase, 95% CI: 131.4 to 1,264.3, p = 0.016). Uncomplicated services did not change at referral Health Centers whereas complicated services increased by 358.1 (32.6% increase, 95% CI: 166.1 to 550.2, p<0.001). All OPD visits did not change. Under-five OPD visits decreased by 192.2 per month (46.1% decrease, 95% CI: -292.9 to -91.6, p = <0.001). There were no statistically discernible changes in ANC1 visits or facility delivery. Patient visits at referral Health Centers increased by 580.1 per month (89.4% increase, 95% CI: 312.6 to 847.5, p<0.001).

### Combined direct (Health Post) and indirect (Health Center) impact of CHA deployment

The total volume of services provided at the Health Post/Health Center pairs was summed in order to analyze the overall effect of CHA deployment on health service provision and patient volumes. Results are summarized in Table 4.

**Table 4:** Total impact of CHA deployment at Health Post and Health Center.

Outcome	Average monthly services at comparison sites		Average monthly services at intervention sites			Difference-in-Difference with controls for staffing, seasonality, and campaigns	
	Baseline	Endline	Baseline	Endline	p	Effect (95% CI)	p
All non-campaign services	7,139	9,505	4,239	7,215	0.003	+848.6 (178.2, 1519.1)	0.013*
Complicated services	2,245	3,216	1,556	2,326	0.424	+416.3 (194.6, 637.9)	<0.001*
Uncomplicated services	4,894	6,288	2,797	4,889	0.001	+432.4 (-111.2, 976.0)	0.118
Specific services							
All outpatient visits	2,062	2,888	1,799	3,149	0.282	-256.3 (-582.5, 69.8)	0.123
Outpatient visits under 5	1,035	1,360	762	1,275	0.563	-215.5 (-347.5, -83.5)	0.002*
Outpatient visits over 5	1,027	1,529	1,037	1,874	0.212	-40.8 (-280.5, 198.9)	0.737
Antenatal care - 1st visit	63.5	66.4	52.1	58.8	0.036	+10.8 (-0.4, 22.1)	0.059
Facility delivery	30.3	35.1	22.5	30.7	0.473	-10.6 (-18.3, -3.0)	0.007*
Patient visits	1,580	1,834	843	1,626	<0.001	+683.3 (389.8, 976.8)	<0.001*

* = significant at the 0.05 level

Combined health service provision at Health Posts and Health Centers increased by 848.6 services per month (20.0% increase, 95% CI: 178.2 to 1519.1, p = .013). The overall volume of complicated services increased by 416.3 (26.8% increase, 95% CI: 194.6 to 637.9, p<0.001) while there was no statistically discernible change in uncomplicated services. No changes in total OPD visits, OPD over 5, and ANC1 visits were statistically discernible. Under-five OPD visits decreased by 215.5 (28.3% decrease, 95% CI: -347.5 to -83.5, p = 0.002) and patient visits increased by 683.3 per month (81.1% increase, 95% CI: 389.8 to 976.8, p<0.001)

### CHA cost-effectiveness and value for investment

The currency conversions use the average conversion rate for 2014 for ZMW to USD. The CHA salaries have subsequently increased such that, in February 2016, the monthly cost for deploying a CHA including salary and amortized training costs is now 3,339 ZMW per month.

The total combined monthly cost for deploying a CHA, including the one-time pre-service training cost amortized over 30 years plus the monthly salary paid by Government Central Payroll, is 2,639 ZMW (431 USD) per month per CHA. When the monthly number of services is divided by the monthly personnel costs the resulting cost effectiveness numbers are displayed in Table 5. The cost-effectiveness numbers below represent the effectiveness of CHAs in directly and indirectly delivering specific services within the health system.

**Table 5:** Relative cost-effectiveness of CHAs at providing additional health services.

Outcome	Cost per service or visit (direct impact*)	Cost per service or visit (indirect impact)	Cost per service or visit (combined impact)
All non-campaign services	-**	ZMW 7.56	ZMW 6.22
Complicated services	-	ZMW 14.74	ZMW 12.68
Uncomplicated services	-	-	-
Specific services:			
All outpatient visits	-	-	-
Outpatient visits under 5	-	(negative impact)	(negative impact)
Outpatient visits over 5	(negative impact)	-	-
Antenatal care - 1st visit	ZMW 573.70	-	ZMW 488.70
Facility delivery	-	-	-
Patient visits	-	ZMW 9.10	ZMW 7.72

* Uses monthly cost of 2369 ZMW per CHA, with two CHAs deployed per Health Post.

** Cost effectiveness was not calculated where no impact was statistically discernible.

## Discussion

This evaluation was designed to measure the direct and indirect impact of CHA deployment on service delivery at rural Zambian health facilities by looking at service and patient volumes at Health Posts where CHAs were deployed and at their referral Health Centers. Based on the combined service delivery data from Health Posts and Health Centers, the deployment of CHAs to rural Health Posts increased the overall number of health services provided as well as the number of patient visits, with much of this impact coming from indirect impacts at referral Health Centers. Thus evaluations that, unlike this one, only measure services directly provided by CHWs may miss an important CHW health system impact.

### Limitations

This study was conducted with the pilot class of Zambia’s CHA cadre. Process evaluations three and six months after CHA deployment [12] highlighted the availability of drugs and commodities and infrequent in-person supervision by off-site supervisors, all of which impacted the acceptability of CHAs to their communities. The CHA program may become more effective as facilities and systems adapt to their deployment. Additional process evaluations of CHA scale-up are planned, and the MOH and Ministry of Community Development, Mother and Child Health (MCDMCH) will continue to refine the program based on evaluation findings.

The main outcomes of this study, volumes of services provided and patient visits, do not take into account the quality of services provided by CHAs. Logistical and budget considerations limited the number of facilities that could be included in this evaluation, resulting in imprecisely estimated effects with large confidence intervals.

Further, while the DiD design of this evaluation separates out the impact of the CHA program from facility characteristics that do not change with time, intervention site selection was an MOH decision rather than randomly selected. The major assumption of DiD analysis is that trends in health services would have been parallel at intervention and comparison facilities in the absence of the intervention, and it is possible that these facilities differed systematically in ways that violate the parallel trends assumption. Budget and logistical constraints meant that data was only collected at 8 health posts and 8 health centers, and while using monthly facility data with facility fixed-effects maximizes the statistical power given these constraints, the standard errors in the analysis are very large. Larger-scale studies exploiting electronic databases of HMIS service delivery data and CHA deployment timing could build on this approach.

Some study sites received additional health workers during the study period; while these were controlled for with dummy variables for the number of clinically trained staff, if the addition of staff resulted in non-linear (i.e., multiplicative) effects then this may confound the analysis. However, more staff were deployed to comparison facilities, so this bias would likely result in underestimated rather than exaggerated effects from CHA deployment.

### Policy relevance

Acknowledging the limitations of this small-scale evaluation, the results of this evaluation have been used by the MOH and MCDMCH to inform decisions about the scale-up of the CHA cadre. A major question for MOH and MCDMCH decision-makers in this changing policy environment is how to establish the appropriate skills mix of health workers given limited resources. The CHA cadre is less expensive to train and employ than nurses and midwives and can be trained and deployed in less than one year. Shifting tasks to lesser-trained, less-expensive cadres like the CHAs, policymakers may rapidly improve access to care with constrained budgets.

Health policymakers work with limited budgets and with many constraints on their choices regarding what type of and how many health workers to deploy. When the CHA cadre was originally conceived, there were larger pay differences between trained health staff such as nurses and midwives and the salary level for CHAs. Payroll reforms in Zambia in September 2013 led to this salary gap being narrowed to ensure CHAs were paid within the range of the revised minimum wage. This evaluation demonstrated the impact CHAs have on health service delivery, but the policy choices around CHA scale-up are driven by both impact and the question of training and deployment costs.

One broader insight of this evaluation is that different cadres of health workers may have complementary effects and should not be evaluated in isolation. The work of CHWs in the community and at rural health facilities may change the mix of services provided at those facilities, or care-seeking patterns at referral health facilities.

In many countries with limited HRH, scaling up highly-skilled clinical cadres to meet the population’s needs is essential, but will require many years. An analysis of the Zambian health workforce published in 2010 [13] found that it would take more than 10 years of large-scale investments in health worker training before Zambia could reach the WHO-recommended minimum of 22.8 health workers per 10,000 population. Shifting tasks to lesser trained CHW cadres may not only fill a gap as they directly provide basic services, but could also maximize the available resources by allowing more highly trained cadres to concentrate on providing more specialized services.
